# Use of Ceramic Membranes in a Membrane Filtration Supported by Coagulation for the Treatment of Dairy Wastewater

**DOI:** 10.1007/s11270-017-3365-x

**Published:** 2017-04-10

**Authors:** Magdalena Zielińska, Maciej Galik

**Affiliations:** Department of Environmental Biotechnology, University of Warmia and Mazury in Olsztyn, Słoneczna Str. 45G, 10-709 Olsztyn, Poland

**Keywords:** Dairy wastewater, Membrane filtration, MF, UF, MF-UF, Coagulation

## Abstract

A membrane filtration system was used to remove organic compounds, suspended solids, colour and turbidity from anaerobically treated dairy wastewater. Direct microfiltration (MF), ultrafiltration (UF), MF-UF and a combination of UF with coagulation using two conventional coagulants were investigated. The installation with ceramic membranes was operated at a pressure of 0.15 MPa (MF) and 0.3 MPa (UF). COD removal was 89 ± 2% in MF, 95 ± 1% in UF and 99% in MF-UF. Apart from size exclusion, removal was also the result of adsorption of organics on the membrane; 3–18% of COD removal was attributed to adsorption. In all these membrane systems, colour removal was 96–98%. Coagulation removed 63–72% of COD at all coagulant doses. In combination with UF, 96–97% of COD was removed. The use of coagulants was ineffective for colour removal; further treatment by UF resulted in above 98% removal. Because of complete rejection of suspended solids, turbidity removal exceeded 99% under all conditions. The use of increased coagulant doses did not have an effect on total efficiency of pollutant removal and on the permeate flux. Coagulation pre-treatment enhanced the performance of filtration only by lengthening the filtration cycle by about 12% as compared to direct UF. Not only was pollutant removal highest in MF-UF, but also the average permeate flux was about 80% higher in this two-stage system than in direct UF. This study shows that the most effective strategy to mitigate membrane fouling is the use of MF as a pre-treatment preceding UF.

## Introduction

In recent years, the production of dairy wastewater in Poland has been about 92,000 m^3^/day (Struk-Sokołowska 2011). Dairy wastewater is characterized by high BOD and COD, due to the presence of dissolved and suspended organic matter. Thus, for dairy processing effluents, the common technologies are anaerobic digestion, valorization for recovery of valuable whey compounds and physicochemical treatment such as membrane separation (Prazeres et al. 2012). Anaerobic digestion offers the advantage of combining pollutant removal and biogas production, with an average methane yield of 0.354 m^3^ CH_4_/kg COD_removed_ (Ramasamy et al. 2004). However, a disadvantage of this process is that the biomass has relatively poor settling properties, which leads to a loss of biomass in the effluent (Lin et al. 2013). Due to the fact that dairy wastewater does not contain toxic chemicals (Sarkar et al. 2006), in recent times, researchers shifted their interests in reuse or recycling of dairy wastewaters to reduce the consumption of fresh water (Riera et al. 2013). If this effluent is to be recycled or discharged from the industrial treatment plant to the local sewage system, the concentrations of total suspended solids (TSS), easily settleable solids and COD must be acceptable.

To produce high-quality effluent for direct reuse in the dairy-wastewater treatment system, membrane filtration is considered a promising method (Kushwaha et al. 2011). Although many studies on dairy wastewater treatment using membrane processes have been performed, the researchers still examine high-pressure or low-pressure membrane techniques. Various membrane techniques have different advantages and disadvantages: Nanofiltration (NF) and reverse osmosis (RO) produce very high-quality permeate (low total organic carbon and conductivity), recover a large volume of permeate (90–95%) (Vourch et al. 2008) and recover lactose and milk proteins (Frappart et al. 2006). Microfiltration (MF) and ultrafiltration (UF) yield a high flux of permeate at low transmembrane pressure (Luo et al. 2011), which means that these techniques have lower energy costs than NF or RO; however, MF and UF reduce COD poorly and do not concentrate small solutes like lactose (Aghili et al. 2016; Chollangi and Hossain 2007). Thus, in most cases, these techniques have been used in pre-treatment for recycling of valuable components like milk proteins (Zhang and Ding 2015).

A problem with membrane filtration alone for treatment of dairy wastewater is that proteinous materials accumulate on the membrane surface, which hampers the direct treatment with membranes (Madaeni and Mansourpanah 2004). To minimize this problem, biological treatment can be combined with separation in anaerobic membrane bioreactors. An external configuration of these bioreactors, which is the most common configuration, has the advantage of easy membrane replacement and high fluxes; however, this configuration needs frequent cleaning and consumes large amount of energy (Le-Clech et al. 2006). In addition, biomass activities in membrane bioreactors can be negatively affected by high cross-flow velocities (Brockmann and Seyfried 1996). Moreover, post-treatment of the effluent with NF was reported to be necessary to produce effluent suitable for recycling (Andrade et al. 2012). To overcome these drawbacks, the membrane installation used in the present study was downstream of the anaerobic bioreactor so that it would reject total suspended solids and COD from the anaerobically treated effluent.

Most of the low-pressure techniques have used polymeric membranes (Chen and Liu 2012; Buntner et al. 2013). However, ceramic membranes have a number of advantages over polymeric membranes. They have good thermal and chemical stability, and high resistance to corrosion, abrasion and fouling, which lead to high efficiency of backwashing and make ceramic membranes more durable (Baker 2000). Also, because of weaker bonding between foulants and the membranes, ceramic membranes can reach much higher flux than polymer membranes (Lee et al. 2013). Thus, although polymeric membranes have lower initial costs, ceramic membranes are worth investigating because their advantages make them more competitive with polymeric membranes over the long term.

There is a growing interest in combining membranes with biological wastewater treatment (Farizoglu and Uzuner 2011). However, the concentration of dissolved pollutants in the permeate can prevent its reuse, which makes it necessary to use the tertiary treatment with high-pressure membrane techniques (Andrade et al. 2014). Although there are some studies concerning filtration of dairy wastewater with ceramic membranes (Farizoglu and Uzuner 2011), there is still limited knowledge about fouling mitigation and removal of COD and suspended solids during post-treatment of anaerobic secondary effluents with tubular membrane modules that contain ceramic membranes. Even with ceramic membranes, fouling remains a problem, which means that membrane filtration is limited by the clogging of membranes with pollutants, which decreases the flux and shortens the filtration cycle and membrane life. The major foulants are proteins that can pass through porous cake layers to create pore blockages in membranes (Yue et al. 2015). One of the methods to control fouling and lengthen membrane life is modification of the feed solution by the use of adsorbents like powdered activated carbon or zeolite as “flux enhancers” (Damayanti et al. 2011). Coagulants can also improve effluent quality and filtration performance; these substances reduce the amount of suspended and colloidal materials responsible for the turbidity of wastewater, thus eliminating organic matter (Ji et al. 2010; Chen and Liu 2012). Although coagulation-membrane filtration is one way to address the problem of fouling, so far it has been more commonly used with raw dairy wastewater (e.g. Formentini-Schmitt et al. 2013) than with anaerobically treated dairy wastewater, even though anaerobic treatment would have the advantage of limiting the volume of chemical sludge produced by coagulation.

In the present study, pressure-driven ceramic membranes were used to post-treat anaerobically treated dairy effluent, with the aim of obtaining permeate with concentrations of COD, TSS, colour and turbidity that would allow the effluent to be discharged to the environment or into a sewage system, or to be recycled for technological processes in the dairy industry. In effluent that can be discharged to the environment, COD should not exceed 125 or 150 mg/L, and TSS should not be more than 35 or 50 mg/L, depending on the size of the wastewater treatment plant (Journal of Laws of Poland, from 2014, item 1800). The maximum pollutant concentrations in the effluent discharged to the sewage system depend on the permissible loading of these pollutants in the wastewater treatment plant (Journal of Laws of Poland, from 2006, No. 136, item 964). The effluent quality that is required for reuse depends on the type of reuse. For example, COD should not exceed 75 mg/L for cooling water or 5 mg/L for steam generation (Andrade et al. 2015). According to the EPA (US Environmental Protection Agency 2012), the concentration of TSS and turbidity should not exceed 0.5 mg/L and 0.2 NTU, respectively, if membranes are used in the filtration process. Although these general requirements do not specify the colour of water, the requirements for different industrial uses depend on the site-specific end use (EPA 2012). This study tested various membrane systems (MF, UF, or MF-UF) to determine the most effective combination of selectivity and permeability when using ceramic membranes for dairy wastewater reclamation. The susceptibility of the ceramic membranes to fouling was investigated, as was the use of coagulation for minimization of fouling.

## Materials and Methods

### Characteristics of Feed Wastewater

The experiments were run with biologically treated dairy wastewater. For biological treatment, model dairy wastewater was prepared from commercial milk powder and contained 33,000 ± 340 mg COD/L, 23,760 ± 260 mg BOD/L, 839 ± 52 mg N/L and 239 ± 37 mg P/L. The biological treatment was conducted under anaerobic mesophilic conditions, at an organic loading rate of 2.1 g COD/(m^3^/day) and a hydraulic retention time of 15.5 days. Biogas production was 360 mL/(g COD_removed_/day). The main characteristics of biologically treated wastewater were 3536 ± 328 mg COD/L, 890 ± 92 mg BOD/L, 1860 ± 220 mg TSS/L, colour of 1.070 ± 0.270, turbidity of 623 ± 140 NTU and 7.3 ± 0.3 pH. This was the inflow into the post-treatment technological system that consisted of membrane filtration or the combination of membrane filtration with coagulation.

### Organization of the Experiment

The experimental set-up is presented in Table 1. In series 1 and 2, MF and UF, respectively, were used as alone processes. In series 3, permeate from MF was exposed to UF. To remove organic compounds, TSS and turbidity, and to increase the hydraulic capacity of the membrane installation, biologically treated wastewater was coagulated and then filtrated by UF (series 4–7). Commercial solutions of 43% (*w*/*v*) ferric sulphate Fe_2_(SO_4_)_3_ and 10% (*w*/*v*) aluminium sulphate Al_2_(SO_4_)_3_ were used as coagulants. Dose 1 (theoretical) of each coagulant was calculated based on the value of wastewater turbidity (T), using Eq. 1 (Kowal and Świderska-Bróż 1996). Dose 1 of 198 mg/L for Fe_2_(SO_4_)_3_ and 181 mg/L for Al_2_(SO_4_)_3_ has been recalculated taking into account the percentage concentrations of coagulants. According to these authors, in the case of simultaneous high intensities of colour and turbidity, the coagulant dosage should be higher. For this reason, in this study, dose 2 was three times bigger than dose 1. The pH of wastewater exposed to coagulation was 7.3, so the pH was not modified. Addition of a coagulant into wastewater was followed by rapid mixing for 30 s at 200 rpm, slow mixing for 20 min at 40 rpm and sedimentation for 30 min. Wastewater after decantation was fed into the membrane installation.

**Table 1:** Organization of the experiment

Series	Process	Coagulant	Coagulant dose
1	MF	–	–
2	UF	–	–
3	MF-UF	–	–
4	Coagulation-UF	Fe_2_(SO_4_)_3_	Dose 1
5	Coagulation-UF	Fe_2_(SO_4_)_3_	Dose 2
6	Coagulation-UF	Al_2_(SO_4_)_3_	Dose 1
7	Coagulation-UF	Al_2_(SO_4_)_3_	Dose 2

1 $$ Dose\ 1=7\sqrt{T}\kern0.5em \left(\mathrm{mg}/\mathrm{L}\right) $$

**Fig. 1:**
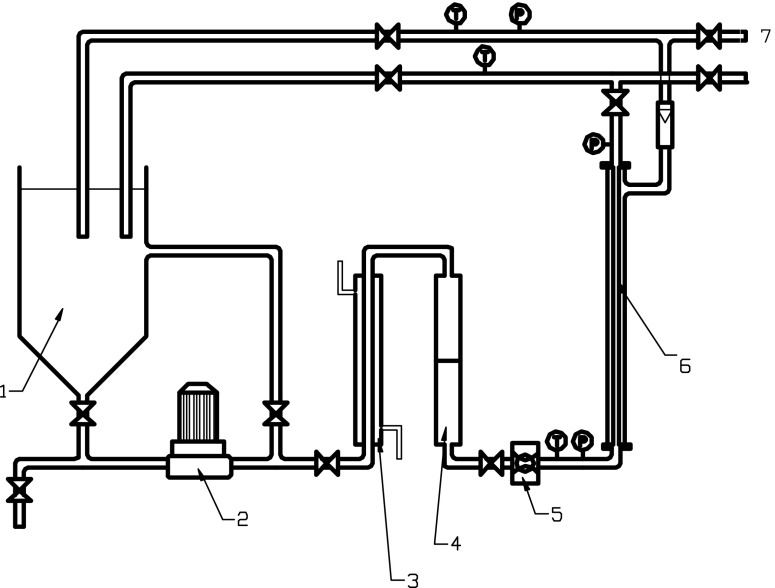
Scheme of the membrane installation: *1* process tank, *2* pump, *3* heat exchanger, *4* prefilter, *5* flow control, *6* membrane module, *7* permeate sampling point, *T* thermometer, *P* manometer

The experiments on membrane filtration of wastewater were conducted in an installation that consisted of a 10-L process tank, a high-pressure pump (CRN(E), Grundfos), a membrane module placed outside the feed tank, a flowmeter, a heat exchanger, a 1-mm prefilter, pressure gauges at the inlet and outlet of the membrane module, a line to circulate the retentate back to the feed tank and a line to receive permeate from the system (Fig. 1). The following streams were introduced to the process tank for membrane filtration: anaerobically treated dairy wastewater (series 1–2), permeate after MF (series 3) or wastewater after coagulation and sedimentation (series 4–7). The membrane module housed one Inside-Céram™ tubular asymmetric ceramic membrane (Tami Industries). This membrane was made from a mixture of TiO_2_ and ZrO_2_. The membrane was 300 mm long with an external diameter of 25 mm. There were 23 channels inside the membrane. Each channel had a hydraulic diameter of 3.5 mm. According to the manufacturer, the total effective filtration area was 0.1 m^2^ and the specific area was 680 m^2^/m^3^. Ceramic membranes for MF (pore size 0.45 μm) or UF (*cut-off* 150 kDa) were used at transmembrane pressure (*TMP*) values typical for particular membrane processes of 0.15 and 0.3 MPa, respectively. During the first filtration cycle in series 3, anaerobically treated dairy wastewater was filtrated with the MF membrane in the membrane module; then in the second filtration cycle, the permeate from MF was filtrated again with the UF membrane in the membrane module. The membrane installation worked under *cross-flow* conditions. The feed solution was pumped into the membrane channels, and the permeate came out from the external membrane walls. To limit membrane clogging, backwashing was periodically done after each filtration cycle, using washing agents recommended by the manufacturer. Each of the series was conducted in duplicate. The presented results are the arithmetic mean of two measurements.

### Membrane Filtration Protocol

Before filtration, the membrane module was flushed by circulating deionized water for 20 min. After that, pure water permeation was measured. The average permeation flux of deionized water (*J*_*W*_) was 37.5 L/(m^2^/min) for the MF and 22.5 L/(m^2^/min) for the UF. Filtrations were performed with a feed flow velocity of 5.2–13.5 L/min and a temperature of 21 °C. The values of these velocities result from the necessity of maintenance of a constant pressure during the process. The retentate was constantly circulated back to the process tank, so these are in fact the velocities of both feed and retentate circulating in the loop throughout the entire time of the process. During filtration, the time necessary for collecting each half of a litre of permeate was measured. These permeation tests were conducted until the membrane was totally clogged and no permeate flow was obtained.

Based on the permeation tests, the permeate flux (filtration rate) (*J*_*V*_, Eq. 2), indicating the transport properties of the membranes, was calculated:

2 $$ {J}_V=\frac{V_P}{t\cdotp A}\kern5.25em \left(\mathrm{L}/\left({\mathrm{m}}^2 \cdot p \mathrm{h}\right)\right) $$

The average permeation flux of deionized water (*J*_*W*_):

3 $$ {J}_W=\frac{V_W}{t\cdotp A}\kern5.25em \left(\mathrm{L}/\left({\mathrm{m}}^2\cdotp \mathrm{h}\right)\right) $$

The efficiency of membrane filtration was determined based on the recovery value, i.e. the fraction of the feed flow which passes through the membrane (*Y*, Eq. 4), the volume concentration factor (*VCF*, Eq. 5) and total membrane resistance (*R*_*m*_, Eq. 6):

4 $$ Y=\frac{V_P}{V_F}\cdot 100\ \left(\%\right) $$

5 $$ VCF=\frac{V_F}{V_F-{V}_P}\left(\hbox{--} \right) $$

6 $$ {R}_m=\frac{TMP}{J_V}\ \left(\left(\mathrm{MPa}\cdot \mathrm{s}\right)/\mathrm{m}\right) $$

The separation properties of the membranes were estimated based on the percentage of rejection (*R*, Eq. 7):

7 $$ R=\left(1-\frac{C_P}{C_F}\right)\cdot 100\ \left(\%\right) $$

The sorption abilities of the membranes were estimated based on the adsorption capacity (*Ads*, Eq. 8):

8 $$ Ads=\left(1-\frac{C_R{V}_R+{C}_P{V}_P}{C_F{V}_F}\right)\cdot 100\ \left(\%\right) $$

The fouling intensity was determined by calculating the normalized permeate flux (*α*, Eq. 9):

9 $$ \alpha =\frac{J_V}{J_W}\ \left(\hbox{--} \right) $$

The abbreviations used in the equations are as follows: *A* membrane filtration area (m^2^), *C*_*F*_ concentration of a pollutant in the feed solution (mg/L), *C*_*P*_ concentration of a pollutant in the permeate (mg/L), *C*_*R*_ concentration of a pollutant in the retentate (mg/L), *t* time for collecting a known volume of permeate (h), *TMP* transmembrane pressure (MPa), *V*_*F*_ volume of feed solution (L), *V*_*P*_ volume of permeate (L), *V*_*R*_ volume of retentate (L) and *V*_*W*_ volume of permeate during filtration of deionized water (L).

### Analytical Methods

To characterize the influent, the wastewater after coagulation, the permeate and retentate after each filtration cycle, COD, TSS, colour, turbidity and pH were measured. COD and TSS were assessed according to APHA (1992). Turbidity was measured with a turbidity meter NANOCOLOR Linus. The pH of wastewater was measured with the use of a HI 2210 pH meter (Hanna Instruments). The colour was calculated using Eq. 10, as a dimensionless value:

10 $$ colour=\frac{{\lambda_{436}}^2+{\lambda_{525}}^2+{\lambda_{620}}^2}{\lambda_{436}+{\lambda}_{525}+{\lambda}_{620}} $$

where *λ* represents the absorbance values measured at three different wavelengths in the visible range (436, 525 and 620 nm) (Bes-Piá et al. 2010). Rayleigh VIS-7220G spectrophotometer was used. In the experimental series with coagulation, the concentrations of iron and aluminium were assessed in wastewater after coagulation and in the permeate spectrophotometrically using LCK320 and LCK301 tests, respectively (Hach Lange GmbH, Germany).

All analyses were performed in triplicate for each sample. The deviation of each measured parameter for each sample was less than 10%.

### Statistical Analyses

Differences between the samples were tested for significance using ANOVA, and the Tukey test after normality and homogeneity of variance was confirmed with the Shapiro-Wilk test and Levene’s test. The strength of the relationships between groups of the results was determined using Pearson’s correlation coefficient. With all statistical analyses, *p* ≤ 0.05 was considered significant. Statistica 10.0 PL (StatSoft) was used.

## Results and Discussion

### Removal of COD, TSS, Colour and Turbidity

**Fig. 2:**
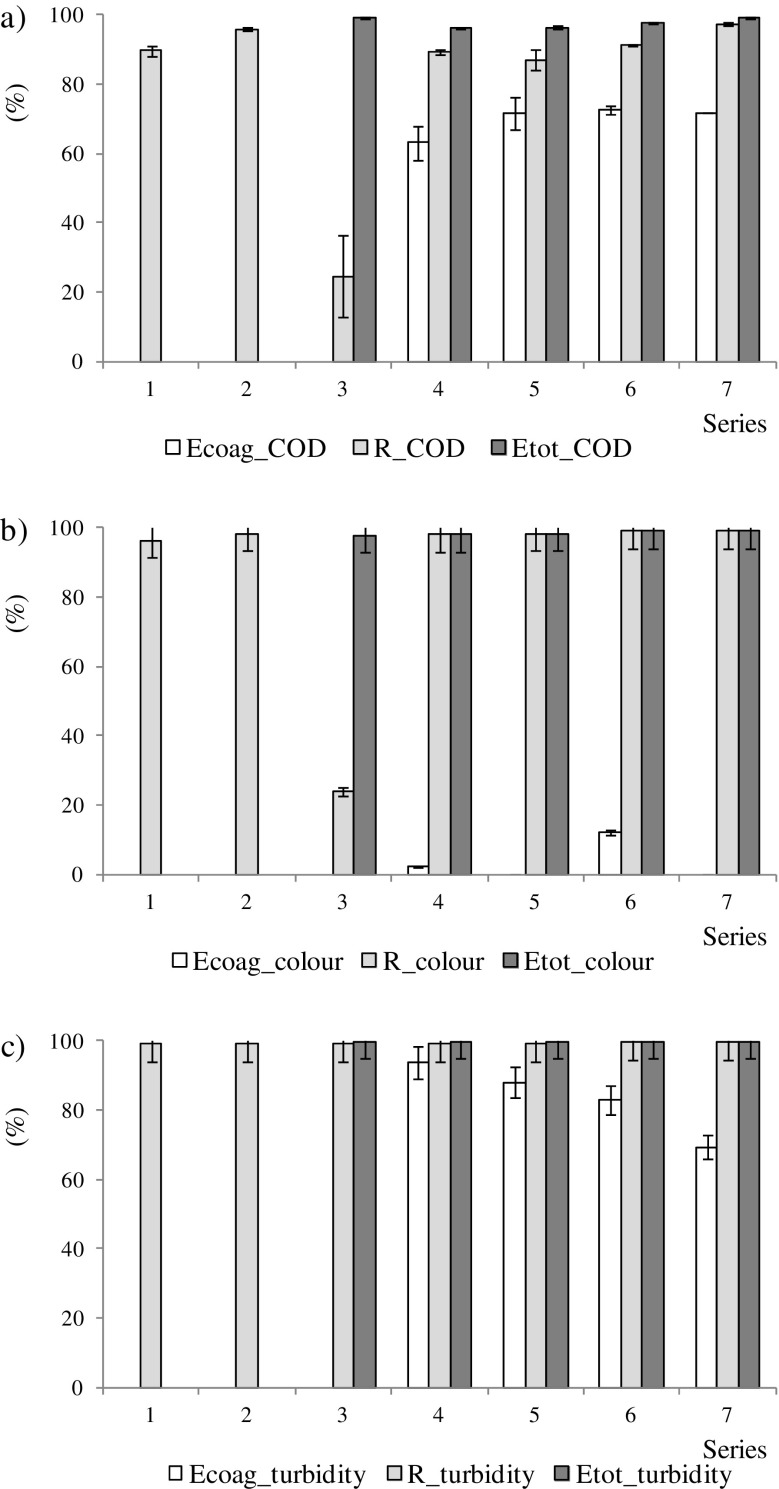
The efficiency of removal in coagulation (*Ecoag*), rejection by a membrane (*R*) and total efficiency in the whole technological system of dairy wastewater post-treatment (*Etot*) for **a** COD, **b** colour and **c** turbidity; in series 3, *R* means rejection by UF—the second stage of membrane filtration

In this study, the efficiencies of membrane filtration alone and in combination with coagulation were evaluated in terms of COD, TSS, colour and turbidity removal. In MF (series 1) and UF (series 2), the average COD concentrations in the permeates were 175 ± 24 and 148 ± 6 mg/L, respectively. The removal of COD with MF was significantly lower than in all other variants. The rejection coefficient (*R_COD*) was 89 ± 2% in MF and increased to 95 ± 1% in UF (Fig. 2a). In MF and UF, the main mechanism responsible for organic matter removal is sieve retention, in which particles are retained on the membrane surface (Guo et al. 2009). The higher COD removal with UF than with MF was due to the lower *cut-off* of the UF membrane; the MF membrane was too open to retain the particles responsible for COD. According to Laabs et al. (2006), organic colloids, polysaccharides and proteins are retained completely by UF membranes and partly by MF membranes. In the present study, in addition, the higher *TMP* in UF (0.3 MPa) than in MF (0.15 MPa) increased the removal of COD because the higher pressure increased the rate of water transport through the membrane and, in consequence, lowered pollutant concentration in the permeate. The use of the two-stage MF-UF system (series 3) was significantly more efficient in COD removal than the use of direct UF. In series 3, UF removed a further 24 ± 11% of COD (*R_COD*), which resulted in total COD removal (*Etot_COD*) of 99%. In series 1–3, total colour removal was 96–98% (Fig. 2b). Thus, the colour was due to particles that were retained even in MF. Because of complete rejection of suspended solids in all investigated membrane systems, turbidity removal by membranes (*Etot_turbidity*) exceeded 99% (Fig. 2c).

Coagulation is an important step in reduction of suspended and colloidal materials, which contribute to turbidity, colour and COD in wastewater. However, in series 4–7, because of the high COD concentration of the dairy anaerobically treated effluent, the physicochemical step of coagulation/flocculation/sedimentation was not sufficient to eliminate the concentrations of pollutants necessary to meet the disposal standards; COD concentrations in wastewater after coagulation were 980–1290 mg/L. This is because not all contaminants can be fully removed by the coagulation process, particularly biopolymers, such as polysaccharides and proteins (Laabs et al. 2006). In the treatment of dairy wastewater, doses of iron and alum coagulant range from 100 to 1000 mg/L. According to Rivas et al. (2010), the optimum reagent dose that was sufficient to eliminate 40–60% of COD and BOD content was 250 mg/L. At a dose of 1000 mg/L, COD removal efficiency was 68 or 62% with alum or ferrous sulphate, respectively (Loloei et al. 2014). The optimum dose was 300 mg/L for alum coagulant and 800 mg/L for iron coagulant, giving 69.2 and 66.5% efficiency of COD removal, respectively (Kushwaha et al. 2010). In the present study, at similar coagulant doses (about 200 and 600 mg/L), the efficiencies of COD removal (*Ecoag_COD*) were 63–72% (Fig. 2a). However, these authors cited above coagulated raw dairy wastewater, whereas in the present study, anaerobically treated dairy wastewater was coagulated. To make it possible to reuse the treated wastewater in the dairy plant or release it into the sewage system or the receiving water bodies, membrane filtration was added as a supplementary step to ensure acceptable effluent quality. In the present study, the membrane system had the capability to maintain high COD removal regardless of the efficiency of coagulation. After coagulation with those two substances, use of UF resulted in 89 ± 1 and 91 ± 1% rejection of the residual COD (*R_COD*), respectively. In these systems, 96–97% of COD were removed, in total, from anaerobically treated dairy effluent. The use of coagulants was ineffective for colour removal; *Ecoag_colour* was 2% for iron coagulant and 12% for alum coagulant (Fig. 2b). After coagulation, UF resulted in 98% colour removal (*R_colour*), independent of the coagulant type, which gave *Etot_colour* of 98–99%. The addition of both coagulants removed turbidity effectively: 93% (iron coagulant) and 83% (alum coagulant). With UF after coagulation (ca. 99% rejection), turbidity was removed almost completely (Fig. 2c). Similarly, direct UF and its combination with pre-treatment by coagulation using *Moringa oleifera* as a coagulant resulted in COD removal exceeding 96% and turbidity and colour removal of 99.9% (Formentini-Schmitt et al. 2013).

Although the efficiency of COD removal in coagulation was significantly higher with the larger dose of iron coagulant (72 ± 5%) than with the smaller dose (63 ± 5%), after UF, the two variants did not differ: *Etot_COD* was 96% with both (Fig. 2a). When using alum coagulant, there was no effect on the *Ecoag_COD* from using a three times higher coagulant dose. Although the use of UF resulted in *Etot_COD* of 96–99%, significantly higher rejection efficiency by the membrane (*R_COD*) was obtained when wastewater was pre-treated with alum coagulant (97 ± 1%) than with iron coagulant (87 ± 3%). With both coagulants, the removal of colour and turbidity in coagulation was less effective at the higher coagulant dose. In the whole system, however, removal of turbidity was almost 100% and that of colour was 98–99% (Fig. 2b, c). To reach turbidity removal of 98.95% in a hybrid system coagulation-membrane bioreactor for reclaiming effluent from the dairy industry, Chen and Liu (2012) determined that the optimum dosage of polyaluminium chloride was much higher (900 mg/L).

To conclude, the highest removal of the evaluated pollutants occurred in MF-UF and in the coagulation-UF systems with the higher dose of aluminium sulphate as a coagulant. However, in order for this system to be widely applied, the residual concentrations in the effluent must be acceptable. According to the Polish law concerning “the highest acceptable concentrations of pollutants from industrial wastewater that are particularly harmful to the aquatic environment”, concentrations of COD in the permeates from MF-UF and from coagulation (both doses of aluminium salt) with UF were acceptable for discharge into the environment. Furthermore, the permeates from all variants were acceptable for discharge into the local sewage system. It should also be remembered that the use of coagulation requires that the amount of iron, aluminium and sulphate in the effluents be controlled. In series 4–7, the iron and aluminium were removed to permissible levels in the whole technological system. Their concentrations did not exceed 10 and 3 mg/L, respectively, which are the highest acceptable values for discharging industrial wastewater to the environment. This indicates that membrane filtration limits the potential threat from undesired ions. Anaerobically treated wastewater itself was the main source of sulphate; the concentration was about 292 mg/L. After coagulation with alum coagulant, it increased by about 70 mg/L (dose 1) and 210 mg/L (dose 2). After coagulation with iron coagulant, it increased by about 100 mg/L (dose 1) and 300 mg/L (dose 2). The types of membranes (MF and UF) used in this study are generally not effective at retaining sulphate. Thus, at higher doses of both coagulants, the concentrations of sulphate in the permeates exceeded 500 mg/L, which is the highest acceptable value for discharging wastewater to the environment.

The coagulation-UF system with both dosages of iron coagulant and with the lower dose of alum coagulant did not remove COD more efficiently than direct UF (series 2). This may be because the particles in the feed solution both without coagulation and after coagulation were larger than 150 kDa, which resulted in high rejection by UF. However, the coagulation-UF system with the higher dosage of alum coagulant (series 7) gave higher total efficiency of COD removal than direct UF (series 2). Because the size of aggregates that are formed during coagulation depends on the coagulant type (Stoller 2009), in our case, the size or amount of the flocs generated during coagulation was larger, which resulted in more effective size exclusion.

The high removal of COD, colour and turbidity even with MF could be explained by the fact that, apart from sieve retention, adsorption is the other mechanism responsible for organic matter removal, in which particles are captured inside the membrane structure, allowing the removal of particles smaller than the pores of the membrane (Guo et al. 2009). In the present study, the mass balance was calculated, based on measured COD loadings in the feed solution, permeate and retentate. These calculations showed that real COD loadings in the retentates were 3–18% lower than theoretical loadings, which indicated that COD removal may have been due to adsorption of organic compounds in the membrane matrix. However, the percentage of organic matter adsorbed was independent of the type of membrane and the pre-treatment conditions, probably because sorption is reversible and there are a limited number of sorption sites on the membrane (Sun et al. 2015).

### Hydraulic Efficiency of Membranes

**Fig. 3:**
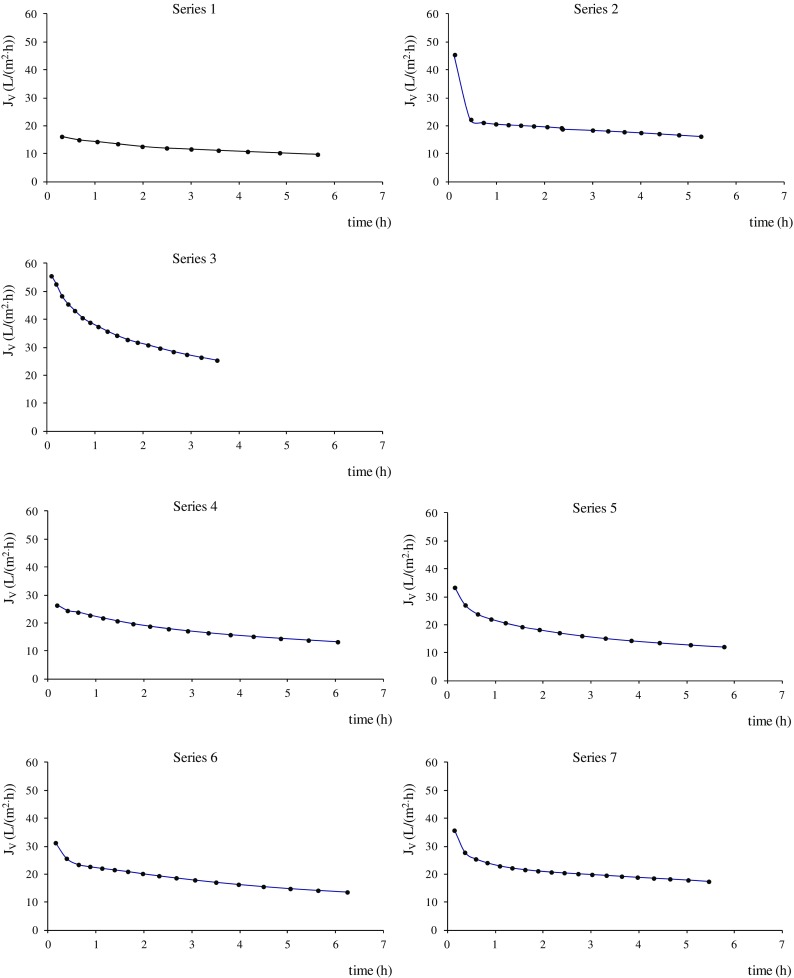
Changes in permeate flux (*J*_*V*_) with time

Together with technological investigations of pollutant removal, this study also determined the most important hydraulic parameters of membrane filtration, such as average permeate flux and membrane resistance. Permeation tests showed that the initial permeate flux was lowest with MF (series 1; Fig. 3). At lower membrane *cut-off*, direct UF resulted in about 2.5 times higher initial flux (*p* = 0.024). This may have been connected with the applied *TMP* (0.15 MPa was used for MF and 0.3 MPa for UF). According to Darcy’s law, an increase in *TMP* generally causes an increase in flux values (Bergamasco et al. 2011; Sun et al. 2015). The MF membrane would need higher pressure to achieve permeate flux similar to the UF membranes. In series 3, wastewater pre-treatment by MF caused a significant increase in the average permeate flux during UF by about 80% (*p* = 0.0001). All experiments were not performed under constant permeate flux, so *J*_*V*_ decreased with time at the beginning of the process. Then, the flux decreased progressively and became stable until the flow stopped (Fig. 3; the final points in these charts were determined just before the permeate stopped flowing). This decrease was caused by blocking of the membrane by pollutants present in the feed solution. When this flow blocking occurred, the membranes were washed. In Table 2, the average values of permeate flux in the whole permeation tests are given. In MF and UF, the mechanism of membrane obstruction must be taken into account in the operation of membrane installations. In the present study, this decrease in the permeate flux indicates fouling, which is an inevitable drawback of the separation process. In MF and UF, this phenomenon is caused by total suspended solids, organic colloids, polysaccharides and proteins that are residual organic materials in secondary effluents and the main contributors to membrane fouling (Laabs et al. 2006). Because TSS are similar in size to the membrane pores in MF, pore blockage is the main fouling mechanism. In addition, fouling results from the concentration polarization, which depends on *TMP*. As the *TMP* increases, more pollutants accumulate on the membrane surface, forming a gel layer and clogging the pores, which increases filtration resistance due to the higher compression of the pollutants (Bergamasco et al. 2011; Sun et al. 2015).

**Table 2:** Hydraulic parameters of membrane filtration

Series	*J*_*V*_ (L/(m^2^/h))	*Y* (%)	*VCF* (−)	*R*_*m*_ ((MPa^.^s)/m)	*α* (−)
1	12.5	42.3	1.7	43,364	0.0055
2	20.5	56.7	2.3	52,570	0.0152
3	36.9	64.3	2.8	19,506	0.0273
4	18.9	50.0	2.0	57,252	0.0140
5	19.0	46.7	1.9	47,493	0.0140
6	19.7	60.7	2.5	54,728	0.0146
7	21.7	67.9	3.1	49,804	0.0161

Although the accumulation of organic matter on the membrane surface decreases the flux, it can help to limit the reduction of rejection efficiency. As a result of fouling, particles forming a cake grow in thickness as the filtration progresses and the effective diameters of membrane pores decrease, which allows for retention of smaller molecules (LaPara et al. 2006). Moreover, colloids that are deposited on the membrane may additionally adsorb dissolved organic compounds, which improves the rejection of pollutants (Andrade et al. 2014). This may explain the fairly high COD rejection in MF in this study, which could have been attributed to both the organic matter particle size and the fouling layer that was formed.

In the present study, a large decrease of volumetric permeate flux in comparison with the flux of deionized water was observed; the value of *α* was lower by an order of magnitude during MF than UF (Table 2). In general, *α* below 1 indicates that the membrane is being fouled by organic matter accumulating in the pores and on the surface of the membrane, which clogs the flux. This was observed in all of the experimental series. However, *α* close to 0 in MF indicates that this membrane tended to become fouled more quickly than the UF membrane. Although fouling is considered to be caused mainly by relatively large colloids and soluble organic compounds ranging from 0.450 to 0.026 μm (Zheng et al. 2009), some authors write that the ratio between pore size and size of the particles is more important in membrane clogging (Lim and Bai 2003). Particles close to or smaller than the pore diameter clog the pores and membrane surfaces and form a filtration cake more quickly and to a greater extent than larger particles. Therefore, in MF, the permeate flux decrease could have been a result of pore blocking and cake formation with larger particles.

In this study, the values for membrane resistance indicate that pore sizes affected the mechanism of permeation. *R*_*m*_ during UF was higher than during MF because of the lower *cut-off* of the UF membrane (Table 2). However, this is not always the case because a membrane with bigger pores is clogged mainly by pollutants that penetrate into the pores. A membrane with smaller pore sizes is clogged mainly by pollutants retained on its surface, and these pollutants could be removed by shearing forces if the filtration is performed in cross-flow mode, as in the present experiment. The MF pre-treatment of the feed solution for UF significantly decreased the membrane resistance to 19,506 (MPa·s)/m, which resulted in the enhancement of permeate flux to 36.9 L/(m^2^/h), the highest obtained value of *J*_*V*_.

In direct UF, adsorbed natural organic matter causes membrane fouling that is not easy to remove and is often irreversible (Guo et al. 2009). However, when UF is combined with coagulation, particles of organic matter are aggregated or sorbed on the flocs of precipitated metal oxides. These particles are deposited on the membrane surface, which constitutes reversible fouling and is easy to clean by physical methods as the filtration progresses. This resistance due to concentration polarization is more easily removed than fouling resistance (Guo et al. 2009). Therefore, in series 4–7 in this study, the combined coagulation-UF process was found to increase membrane lifetime. The permeate flux during UF was significantly higher than during MF, which indicated that the UF membrane has higher capacity. For this reason, in this study, the UF membrane was considered more promising because it obtained higher fluxes, and the effect of coagulation on the flux through only this membrane was examined. The flow stoppage was observed about 2 h later than during direct UF (Fig. 3). Lower frequency of membrane washing will lower the operational cost. However, the initial *J*_*V*_ and the average *J*_*V*_ values were similar to those obtained during direct UF and significantly lower than that obtained during MF-UF (Table 2) (*p* = 0.0001). According to Stoller (2009), coagulation should significantly reduce fouling due to reduction of solute concentration by sedimentation and particle size shifts. However, different coagulants give different particle size shifts. The diameters of the aggregates produced in coagulation should not be similar to the pore size.

In the present study, although the increase in the coagulant dose resulted in a decrease in membrane resistance, it did not have an effect on the permeate flux; *J*_*V*_ was from 18.9 to 21.7 L/(m^2^/h) (Table 2). Although a critical coagulant dose could cause dramatic flux reduction by blocking the membrane pores after coagulating (Ma et al. 2014), in the present study, the higher coagulant dose did not affect the recovery value and the volume concentration factor. These are important parameters for the economic viability of the membrane process when planning to reuse water. Independently of the operational conditions, *Y* was from 42.3 to 67.9%. In addition, the intensity of fouling measured as the normalized permeate flux was similar during direct UF and combined coagulation-UF. Based on the results of pollutant rejection and hydraulic capacity of the membranes, it can be concluded that there is no necessity to increase the coagulant dose above the theoretical dose.

## Conclusions

This paper examined the applicability of membrane filtration to reclaiming anaerobically treated dairy wastewater. The results indicated that the ceramic membranes had high rejection efficiency: >87% of COD was removed, as was >96% of colour, and almost all TSS and turbidity. Coagulation did not influence the total removal of pollutants in the systems and was not a factor that controlled membrane fouling effectively. The most important advantage of MF-UF is that the low-pressure membranes can remove COD and turbidity almost completely. This two-stage configuration was the most useful of those tested for treatment of dairy secondary effluent, based on both rejection efficiency and flux capacity. The outcome of this study could be useful in the development of post-treatment of dairy wastewater for reuse purposes.
